# Early and late preventive effect of *Nigella sativa *on the bleomycin-induced pulmonary fibrosis in rats: An experimental study

**Published:** 2018

**Authors:** Hamid Reza Poursalehi, Mitra Samareh Fekri, Fariba Sharifi Far, Ali Mandegari, Atefe Izadi, Rahil Mahmoodi, Hadi Nematollahi, Fateme Porgholamhosein, Vahideh Ghorani, Masome Samareh Fekri

**Affiliations:** 1 *Physiology Research Center of Kerman University of Medical Sciences, Kerman, Iran*; 2 *Cardiovascular Research Center, Institute of Basic and Clinical Physiology Sciences, Kerman University of Medical Sciences, Kerman, Iran*; 3 *Department of Pharmacognosy, Faculty of Pharmacy, Kerman University of Medical Sciences, Kerman, Iran*; 4 *Department of Toxicology and Pharmacology, Faculty of Pharmacy, Kerman University of Medical Science, Kerman, Iran*; 5 *Department of Pharmacy, Kerman University of Medical Science, Kerman, Iran*; 6 *Herbal & Traditional Medicines Research Center, Faculty of Pharmacy Kerman University of Medical Sciences, Kerman, Iran*; 7 *Neurogenic Inflammation Research Center and Department of Physiology, School of Medicine, Mashhad University of Medical Sciences, Mashhad, Iran*; 8 *Assistant Professor of Plant Protection, Department of agriculture*

**Keywords:** Bleomycin, Hydroxyproline, Nigella sativa, Pulmonary inflammation Fibrosis

## Abstract

**Objective:**

Pulmonary fibhrosis is a disease of the connective tissues in the respiratory system. *Nigella sativa *has been used for the treatment of pulmonary diseases like asthma. This study investigated the early and late preventive effect of methanolic extract of *N. sativa* on a bleomycin- induced pulmonary fibrosis model.

**Materials and Methods:**

This study was carried out using 52 rats. Pulmonary fibrosis was induced by a single endotracheal injection of bleomycin (5 mg/kg). Extract of *N. sativa* (500 mg/kg per day) or methylprednisolone succinate (4 mg/kg per day) was injected intraperitoneally in two periods (i.e. days 1-14 as early preventive group and days 15-28 days as late preventive group). The lung tissues were histologically examined at the end of each period and inspected for the amount of hydroxyproline and biomarkers of oxidative stress.

**Results:**

The pulmonary inflammation and fibrosis were significantly decreased in groups treated with methylprednisolone and *N. sativa* extract compared to bleomycin group in both early and late prevention groups (p<0.001). The hydroxyproline concentration in pulmonary tissue was significantly decreased in *N. sativa* and methylprednisolone groups compared to the bleomycin group in both prevention groups (p<0.001). Significant reductions in lipid peroxidation (p<0.001) and increases in catalase activity were also observed in *N. sativa *and methylprednisolone groups compared to bleomycin group.

**Conclusion:**

This study suggested that* N. Sativa* extract is effective for early and late prevention of pulmonary fibrosis and inflammation. However, more studies are needed to identify its anti-inflammatory and anti-fibrotic mechanisms in the respiratory system.

## Introduction

Pulmonary fibrosis is a disease of the connective tissues in the respiratory system which begins with airspaces damage and continues with the inflammation and accumulation of collagen and extracellular matrix in the airspace walls. The damage stimulates epithelial and endothelial cells and leads to the accumulation of inflammatory cells in the airspaces. Free radicals are then released and they result in the transudation of different cytokines. These agents stimulate fibroblast proliferation in the airspaces which ultimately leads to collagen deposition in the airspace walls (Chen et al., 2006 ▶; Demedts and Costabel, 2002 ▶; Kim et al., 2006 ▶). In some cases, the cause of this disease is known, but in cases of unknown cause, it will be referred to as idiopathic pulmonary fibrosis (IPF) (Chen et al., 2006 ▶; Demedts and Costabel, 2002 ▶). Viral and bacterial infections, damage from some mineral compounds and side effects of some chemical medicines such as bleomycin (BLM) and methotrexate, are among the causes of pulmonary fibrosis (Kuwano et al., 2001 ▶). BLM side effects include lung structure damages which are reflected by increased hydroxyproline and collagen deposition in the lung (Azambuja et al., 2005 ▶). 

Oxidative stress is one of the main mechanisms involved in pathogenesis of pulmonary fibrosis (Kinnula et al., 2005 ▶). Oxidative stress in biological systems was animbalance between oxidants and antioxidants in favor of oxidants which potentially leads to cell damage (Sies, 1985 ▶; Spatz and Bloom,1992 ▶; Knight, 1998 ▶). Existing evidence shows that oxidative stress has been implicated in over 100 different diseases (Pincemail, 1995 ▶).

*N. sativa* is a grassy plant belongs to the Ranunculaceae family‚ which has beenwidely used in the Middle East, India and North Africa. There are many reports concerning the biological and pharmacological activity of this plant, such as immunomodulatory, anti-inflammatory, pain alleviating, antidiabetic, antibacterial, antifungal, anticancer, antioxidants and anti-hypertensive effects (Ashraf et al., 2011 ▶; Ghannadi et al., 2005 ▶; Saad, 1975 ▶; Zaher et al., 2008 ▶). Protective effect of *N. sativa *on kidney and liver injury which is mediated through its antioxidant properties, is due to the presence of polyphenolic compounds. *N. sativa *has shown protective effects on lung injury induced by sulfur mustard in guinea pigs and it has shown therapeutic effects in asthmatic patients without having toxicity (Al Ameen et al., 2011 ▶; Aziz Dollah et al., 2013 ▶; Danladi et al., 2013 ▶; Dollah et al., 2013 ▶; Hossein et al., 2008 ▶; Onoshe and Madusolumuo, 2014 ▶).

Thymoquinone‚ the main component of *N. sativa, *decreases tracheal response, diminishes the number of inflammatory cells in bronchial lavage fluid and reduces tracheal smooth muscle contraction in ovalbumin and methacholine-sensitized guinea pigs (Boskabady et al., 2011b ▶; Pejman et al., 2014 ▶).

In a review article, bronchodilatory, anti-hypertensive and antispasmodic effects of *N. sativa* as well as the underlying mechanisms of its relaxant effects on the airways smooth muscles and vascular, gastrointestinal and urogenital systems were reported (Keyhanmanesh et al., 2014a ▶).

 Since there is no conclusive evidence to support the antifibrotic effect of this herb, the present study investigated the early and late preventive effect of methanolic extract of *N. sativa *on BLM-induced pulmonary fibrosis in rats.

## Materials and Methods

**Plant materials**

*N. sativa* was collected from Koohpayeh area, Kerman, Iran in June 2013. A voucher specimen was kept in the herbarium center of faculty of pharmacy. Seeds of the plant were artificially dried in an oven at < 40°C. The seeds were milled and passed through a sieve number 300. Methanolic extract of *N. sativa *was prepared using the percolation method. The extract was concentrated by rotary apparatus, dried in an oven and kept at -20°C until initiation of the experiment.

The present study was performed on 52 male albino rats weighing 180-200 g. Animals were kept at 20-22°C with12hr/12hr dark-light cycle. They had free access to food and water. Since LD50 value of the plants in rats was higher than 1g/kg body weight (Gali-Muhtasib et al., 2006 ▶), in the present study *N. sativa *was used with a safe dose of 500 mg/kg. All procedure and animal care were approved by the Ethics Committee of Kerman University of Medical Sciences (Permission No 92/146KA).

**Experimental groups**

The study was performed in two conditions as a) Early prevention groups (early P. group) which animals received M-pred and *N. sativa* extract or saline (in control and BLM groups) intraperitoneally from day 1 to day 14 and b) Late prevention group (late P. group) which animals were treated with the same agents from day 15 to day 28. In each condition, animals were randomly allocated to the following groups:

(1) Saline (Control) group: the animals received normal saline (0.9% intratracheally) (n=4 for early prevention group and n=6 for late prevention group).

(2) BLM group: the animals received BLM (5 mg/kg body weight, intratracheally) (n=7 for early prevention group and n=5 for late prevention group).

(3) BLM+ methylprednisolone sodium succinate (M-pred): the animals received the M-pred (4 mg/kg, intraperitoneal) once a day (n=7for both early and late prevention groups). 

(4) BLM+ *N. sativa* extract: the animals received *N. sativa* extract, 500 mg/kg, intraperitoneally (n=8 for both early and late prevention groups). 

The number of animals was determined based on a previous study (Gali-Muhtasib et al., 2006 ▶).

**Experimental design**

The animals were anesthetized using ketamine; then a single dose of BLM (5mg/kg body weight) was intratracheally injected into the lungs of animals in the test groups. The control group intratracheally received a single equal volume of normal saline 0.9% into the lungs (Chen et al., 2012 ▶; Liang et al., 2011 ▶; SamarehFekri et al., 2013 ▶). Subsequently, saline and BLM groups intraperitoneally received saline once a day for a duration of 2 weeks. M-pred and *N. sativa *groups intraperitoneally received drugs once a day from day 1 to day 14 after injection of a single endotracheal dose of BLM (early P. groups). However, late P. groups received M-pred and *N. sativa *once a day from day 15 to day 28 after BLM administration (Chen et al., 2012 ▶; Zhou et al., 2007 ▶). On the 14^th^and 28^th^ day, for investigating the early and late preventive effects on pulmonary fibrosis and inflammation, lung was removed and fixed in formalin solution and the remaining parts were frozen in liquid nitrogen and then maintained at -80^◦^C to measure hydroxyproline concentration (Chen et al., 2012 ▶; Woessner Jr, 1961 ▶). 

The samples were coded with numbers by the table of random numbers (so the pathologists were unaware of the animal grouping). Next, the tissues were cut (5-µm sections) by a microtome (IEICA, Germany) and stained with hematoxylin-eosin (H&E) and Masson’s trichrome methods. Only one pathologist evaluated and scored the samples and investigated inflammatory infiltrates (Sur et al., 1999 ▶) and tissue fibrosis (Ashcroft et al., 1988 ▶) based on the following scale.

**Tissue inflammation**

0: Without inflammation 

1: Existence of local inflammatory cells

2: The majority of the bronchi and veins are surrounded by a thin layer of inflammatory cells (thickness of 1-5 cells)

3: The majority of the bronchi and veins are surrounded by a thick layer of inflammatory cells (a thickness of more than 5 cells).

4: Complete pulmonary inflammation around all veins and bronchi (Sur et al., 1999 ▶).

**Tissue fibrosis**

0: Normal pulmonary tissue

1: Minimal thickness in alveoli and bronchiole walls

2-3: Medium thickness in alveoli and bronchiole walls without significant damage to lung structure

4-5: Increase in fibrosis with significant damage to lung structure and formation of small clumps of fibrosis

6-7: Severe destruction of structure, large areas of fibrosis and “honeycomb lung”

8: Total fibrosis domination over lung (Ashcroft et al., 1988 ▶).

**Hydroxyproline assay**

Hydroxyproline (HYP) was assessed to measure collagen deposition in the lung (Reddy and Enwemeka, 1996 ▶). For this purpose, 50-100 mg of the lung tissue was homogenized in 1 ml PBS (phosphate buffered saline) by Ika-T18B homogenizer (Germany) and centrifuged for 20 minutes in Nuve – NF800R refrigerated centrifuge (Turkey) at 2500 RPM. Then, supernatant was isolated for assessing HYP concentration. Rat HYP ELISA kit was employed following company's instructions (Aoki et al., 2005 ▶). The values were expressed as HYP content per protein content of the samples.

**Determination of oxidative stress biomarkers in lung homogenized tissues**

Total antioxidant capacity was determined based on the methods suggested by Benzie, Strain, and Mandegari. Lipid peroxidation was determined based on the methods suggested by Esterbauer, Cheeseman, and Mandegari. Catalase activity was evaluated by its ability to break down hydrogen peroxide into water and oxygen using spectrophotometry based on Beers and Sizer’s method (Beers and Sizer, 1952 ▶; Esterbauer and Cheeseman, 1990 ▶; Mandegary et al., 2012 ▶). 

**Statistical analysis**

Data were reported as mean±SD. Having proved the normality of data, ANOVA test was used to show the differences in the analyzed indices. Gabriel test was used to perform within group comparisons and SPSS Ver. 20 was employed for data analysis. P values < 0.05 were considered as significant (p<0.05).

## Results

**Effect of** ***N. sativa*** **extract on the pathologic pulmonary inflammation**

*Inflammation scores*

The results of pathological evaluations in early P. groups showed that 75% of rats in the saline group had an inflammation score of 0 to 1 while in the BLM group‚ inflammation score for all rats was 2 to 3. In the M-pred group, 71.4% of rats indicated an inflammation score of 2 while in *N. sativa *group, inflammation score for 75% of rats was 1 (Table 1). Regarding the results of late P. groups, in the saline group, 100% of rats had an inflammation score of 0 to 1 while in the BLM group‚ inflammation score for all rats was 2 to 3. In addition, 71.4% of rats in the M-pred group had an inflammation score of 2 to 3 while inflammation score of all rats in the *N. sativa *group was 1 to 2 (Table 2). 

**Table 1:** Pulmonary inflammation scores in early prevention groups (on the 14^th ^day).

	**Inflammation scores**				
	**0**	**1**	**2**	**3**	**n**
**Saline**	2^a^ (50%)	1 (25%)	1 (25%)	0 (0%)	4
**BLM**	0 (0%)	0 (0%)	4 (57.1%)	3 (42.9%)	7
**M-pred**	0 (0%)	2 (28.6%)	5 (71.4%)	0 (0%)	7
***N. sativa***	0 (0%)	6 (75%)	2 (25%)	0 (0%)	8

Data were shown by a: numbers of rats. n=number of rats each group. Statistical comparisons were made using Chi-Square test. BLM: Bleomycin; Saline: Normal saline; M-pred: Methyl prednisolone; *N. sativa: Nigella Sativa*

**Table 2:** Pulmonary inflammation scores in late prevention groups (on the 28th day

	**Inflammation scores**				
	**0**	**1**	**2**	**3**	**n**
**Saline**	3^a^ (50%)	3 (50%)	0 (0%)	0 (0%)	6
**BLM**	0 (0%)	0 (0%)	3 (60%)	2 (40%)	5
**M-pred**	0 (0%)	2 (28.6%)	4 (57.1%)	1 (14.3%)	7
***N. sativa***	0 (0%)	5 (62.5%)	3 (37.5%)	0 (0%)	8

Data were shown by a: numbers of rat. n=number of rats each group. Statistical comparisons were made using Chi-Square test. BLM: Bleomycin; Saline: Normal saline; M-pred: Methyl prednisolone; *N. sativa: Nigella Sativa*

**Inflammation severity**

Evaluation of pulmonary inflammation severity in studied groups showed that the average pulmonary inflammation was significantly increased in BLM group compared to saline group (p<0.001) in both early (on the 14^th^ day) and late(on the 28^th^ day) P. groups. In addition, average pulmonary inflammation in the *N. sativa* group, similar to M-pred group was significantly lower than that of the BLM group in both early and late P. groups (p<0.001)(Table 3).

**Effect of** ***N. sativa*** **extract on the pathologic pulmonary fibrosis**

*Fibrosis scores*

Evaluation of pulmonary fibrosis in early P. groups showed that in the saline group, 100% of rats had a fibrosis score of 0 to 1 while in the BLM group, fibrosis score for 71.4% of rats was of 4 to 7. In the M-pred group, 28.6% of rats showed a fibrosis score of 0 to 1 while in *N. sativa *group, fibrosis score for 87.5% of rats was 0 to 1 (Table 4). In late P. groups, 100% of rats in the saline group had a fibrosis score of 0 to 1 while in the BLM group, fibrosis score for 80% of rats was 4 to 7. Additionally, fibrosis score in the M-pred group for 100% of rats was 1 to 3 while in the *N. sativa *group, all rats indicated a fibrosis score of 0 to 1 (Table 5). 

**Table 3:** Pulmonary inflammation severity in early prevention groups (on the 14^th ^day) and late prevention groups (on the 28^th ^day

	**Inflammation** **in early P. group**	**n**	**Inflammation** **in late P. group**	**n**
**Saline**	0.77±0.47*	4	0.6±0.24*	6
**BLM**	2.6±0.24	7	2.6±0.24	5
**M-pred**	1.57±0.20*	7	1.57±0.20*	7
***N. sativa***	1.14±0.14*	8	1.28±0.48*	8

Values are presented as mean±SD. n=number of rats each group. Statistical comparisons were made using ANOVA and Gabriel test.

* p<0.001 shows significant differences compared to BLM group. BLM: Bleomycin; Saline: Normal saline; M-pred: Methyl prednisolone; *N. sativa: Nigella Sativa*.

**Table 4:** Pulmonary fibrosis scores in early prevention groups (on the 14^th^ day).

	**Fibrosis scores**					
	**0**	**1**	**2-3**	**4-5**	**6-7**	**n**
**Saline**	2^a^ (50%)	2 (50%)	0 (0%)	0 (0%)	0 (0%)	4
**BLM**	0 (0%)	0 (0%)	2 (28.6%)	4 (57.1%)	1 (14.3%)	7
**M-pred**	0 (0%)	2 (28.6%)	5 (71.4%)	0 (0%)	0 (0%)	7
***N. sativa***	1 (12.5%)	6 (75%)	1 (12.5%)	0 (0%)	0 (0%)	8

Data were shown by a: numbers of rat. n=number of rats each group. l comparisons were made using Chi-Square test. BLM: Bleomycin; Saline: Normal saline; M-pred: Methyl prednisolone; *N. sativa: Nigella Sativa*.

**Fibrosis severity**

Assessment of pulmonary fibrosis severity in studied groups demonstrated that the average pulmonary fibrosis in BLM group was significantly increased compared to saline group (p<0.001) in both early (on the 14^th^ day)and late(on the 28^th ^day) P. groups. Average pulmonary fibrosis in groups treated with *N. Sativa *and M-pred was significantly lower than that of BLM group in both early and late P. groups (p<0.001for both cases), (Table 6).

**Table 5:** Pulmonary fibrosis scores in late prevention groups (on the 28^th^ day).

	**Fibrosis scores**					
	**0**	**1**	**2-3**	**4-5**	**6-7**	**n**
**Saline**	4^a^ (66.7%)	2 (33.3%)	0 (0%)	0 (0%)	0 (0%)	6
**BLM**	0 (0%)	0 (0%)	1 (20%)	3 (60%)	1 (20%)	5
**M-pred**	0 (0%)	4 (57.1%)	3 (42.9%)	0 (0%)	0 (0%)	7
***N. sativa***	3 (37.5%)	5 (62.5%)	0 (0%)	0 (0%)	0 (0%)	8

Data were shown by a: numbers of rat. n=number of rats each group. Statistical comparisons were made using Chi-Square test. BLM: Bleomycin; Saline: Normal saline; M-pred: Methyl prednisolone; *N. sativa: Nigella Sativa*

**Table 6:** Pulmonary fibrosis severity in early prevention groups (on the 14^th ^day) and late prevention groups (on the 28^th ^day

	**Fibroses** **in early P. group**	**n**	**Fibroses** **in late P. group**	**n**
**Saline**	0.5±0.28*	4	0.4±0.24*	6
**BLM**	3.16±0.40	7	3±0.31	5
**M-pred**	1.71±0.18*	7	1.28±0.18*	7
***N. sativa***	0.85±0.14*	8	0.57±0.20*	8

Values are presented as mean±SD. n=number of each group. Statistical comparisons were made using ANOVA and Gabriel test.

* p<0.001shows significant differences compared to BLM group. BLM: Bleomycin; Saline: Normal saline; M-pred: Methyl prednisolone; *N. sativa: Nigella Sativa*.

**Effect of** ***N. sativa*** **extract on lung hydroxyprolineconcentration**

The results of hydroxyproline concentration (µg/l) in early P. groups showed that the average hydroxyproline concentration in BLM group (0.043±0.01µg/l) was significantly higher than saline group (0.019±0.001µg/l). The average concentration was significantly reduced in groups treated with *N. sativa *(0.023±0.006µg/l) and M-pred (0.022±0.007µg/l) compared to BLM group (Table 7). 

Similarly, evaluation of hydroxyproline concentration in late P. groups showed that the average concentration in BLM group was (0.048±0.01µg/l) which was significantly higher than that of the saline group. This concentration was significantly reduced in the *N. sativa *(0.023±0.006µg/l) and M-pred (0.026±0.006µg/l) groups compared to BLM group (Table 7).

**Table 7:** Hydroxyproline concentration (µg/l) per protein content (mg) in early (on the 14^th^ day) and late (on the 28^th^ day) prevention groups

	**hydroxyproline** **Early P. group**	**n**	**Hydroxyproline** **Late P. group**	**n**
**Saline**	0.019±0.001*	4	0.027±0.009*	6
**BLM**	0.043±0.01	7	0.048±0.01	5
**M-pred**	0.022±0.007*	7	0.026±0.006*	7
***N. sativa***	0.023±0.006*	8	0.023±0.006*	8

Values are presented as mean±SD. n=number of rats each group. Statistical comparisons were made using ANOVA and Gabriel test.

* p<0.001shows significant differences compared to BLM group. BLM: Bleomycin; Saline: Normal saline; M-pred: Methyl prednisolone; *N. sativa: Nigella Sativa*.

**Effect of** ***N. sativa*** **extract on lipid peroxidation and catalase activity**

**Figure 1:**
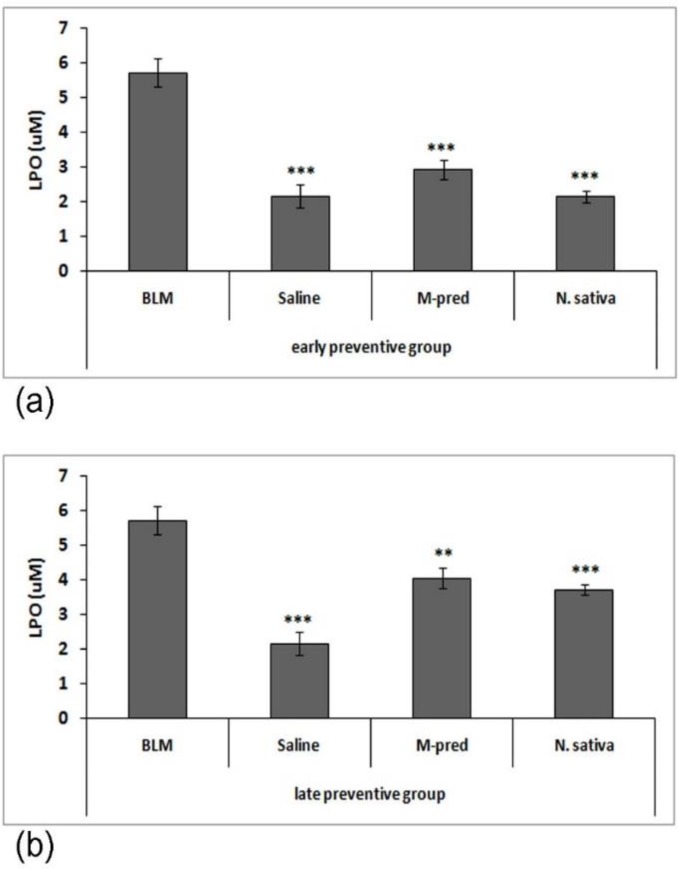
Early (a) and late (b) preventive effect of *N. sativa *methanolic extract on lipid peroxidation (LPO) in bleomycin group (BLM, n=7 for early P. groups and n=5 for late P. groups), normal saline group (Saline, n=4 for early P. groups and n=6 for late P. groups), BML-treated with methyl prednisolone (M-pred, n=7) and *N. sativa* (*N. sativa*, n=8). Data are presented as mean±SD values. Statistical comparisons were made using ANOVA and Gabriel test. **p<0.01 and ***p<0.001 show significant differences vs. BLM group

Assessment of lipid peroxidation in the homogenized pulmonary tissues showed that level of lipid peroxidation in BLM group was significantly higher than that of saline group (p<0.001). On the other hand, the level of lipid peroxidation in M-pred and *N. sativa* groups was significantly reduced compared to BLM group in both early (p<0.001) and late (p<0.01 to p<0.001) P. groups. However, this reduction in early P. groups was higher than late P. groups (Figure 1).

**Figure 2:**
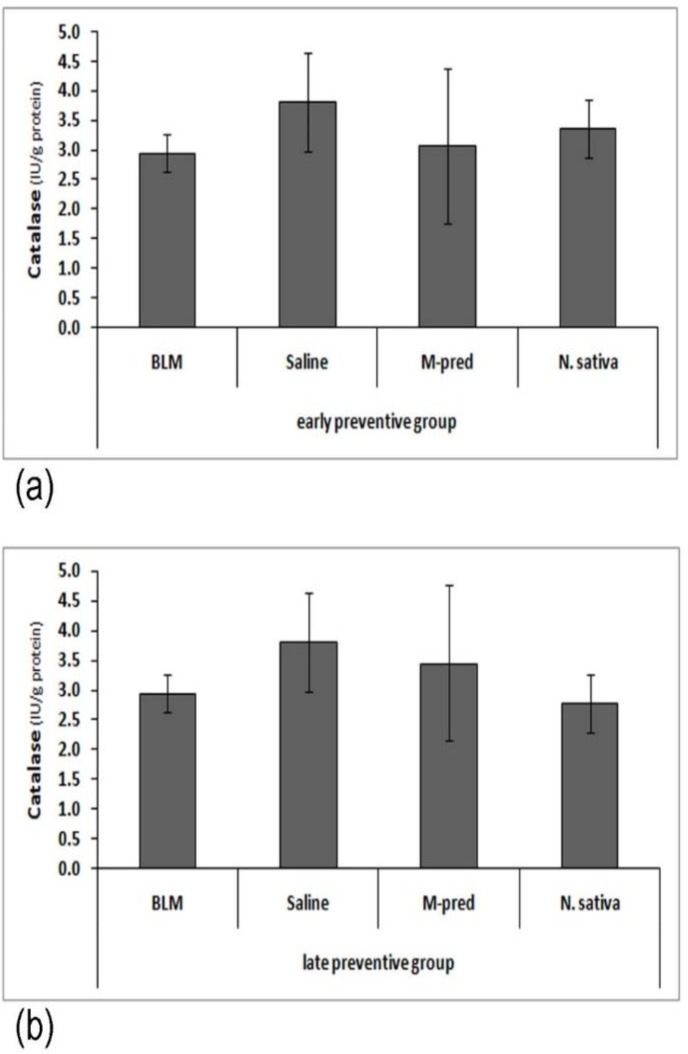
Early (a) and late (b) preventive effect of *N. sativa *methanolic extract on catalase activity in bleomycin group (BLM, n=7 for early P. groups and n=5 for late P. groups), normal saline group (Saline, n=4 for early P. groups and n=6 for late P. groups), BML-treated with methyl prednisolone (M-pred, n=7) and *Nigella sativa* (*N. sativa*, n=8). Data are presented as mean±SD. Statistical comparisons were made using ANOVA and Gabriel test

Additionally, evaluation of catalase activity in both early and late P. groups indicated a reduction in catalase activity in BLM group compared to saline group; however, this difference was not statistically significant. In groups treated with *N. sativa* and M-pred, catalase activity was non-significantly increased compared to BLM group (Figure 2).

**Effect of** ***N. Sativa*** **extract on BLM-induced pulmonary histopathology**

**Figure 3:**
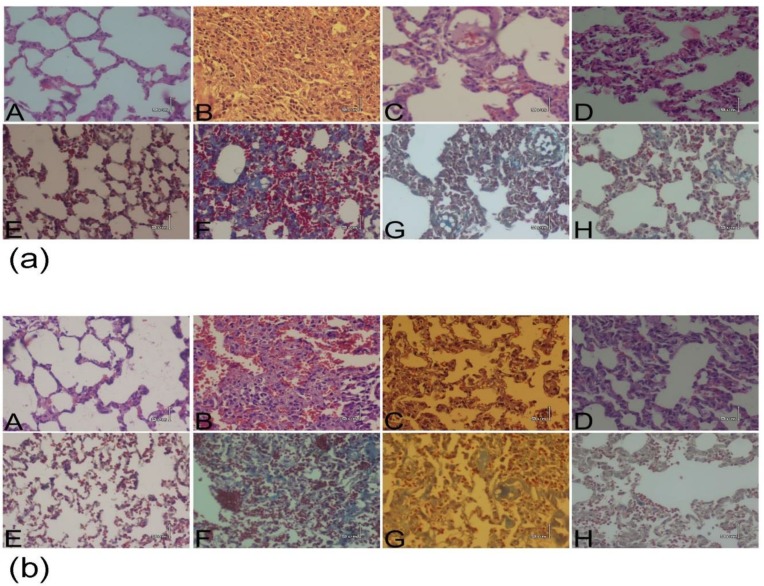
BLM-induced lung histopathological changes in early (a) and late (b) P. groups. The top pictures (A-D) taken following hematoxylin-eosin staining for pulmonary inflammation and the bottom pictures (E-H) are related to Masson‘s trichrome staining method for depicted pulmonary fibrosis. A, E: Saline group; B, F: BLM group; C, G: M-pred group; D, H: *N. sativa* group. (Magnification X100). BLM: bleomycin; Saline: normal saline; M-pred: methyl prednisolone; *N. sativa*: *Nigella sativa*

There was normal wall thickness and alveolar space in saline group while in BLM group, infiltration of inflammatory cells mainly lymphocytes and neutrophils, was observed. There was also fibrotic changes in the lung including thickening of alveolar/bronchiole, alveolar space collapse, fibroblast proliferation and replacement of extracellular matrix with inflammatory cells in BLM group. However, inflammation and fibrosis was improved in groups treated with M-predand *N. sativa *(Figure 3).

## Discussion

This study investigated the effect of methanolic extracts of *N. sativa *on the early and late prevention of BLM-induced pulmonary fibrosis in rats. This study showed that pulmonary inflammation and fibrosis in BLM group were significantly higher than those of saline group in early (on the 14^th^ day) and late (on the 28^th^day) P. groups while, pulmonary inflammation and fibrosis in *N. sativa *group similar to M-pred group were significantly decreased compared to BLM group. Both *N. sativa *and M-pred groups showed a significant decrease in hydroxyproline concentration, an index of collagen deposition, compared to BLM group. The level of lipid peroxidation in lung tissues was significantly decreased in M-pred and *N. sativa* groups compared to BLM group; however, this reduction was higher in *N. sativa *group than M-pred group. Catalase activity was also increased in *N. sativa *group similar to M-pred group but it was not statistically significant. These results indicated that the effect of *N. sativa *extract is comparable to that of M-pred. Based on these observations, *N. sativa* can be suggested as an anti-inflammatory and anti-fibrotic drug which acts via decreased lipid peroxidation level and increased tissue catalase production.

Idiopathic pulmonary fibrosis (IPF) is a chronic, incurable and disabling pulmonary disease which presents destruction of pulmonary tissue causing progressive dyspnea. The etiology of this disease is mostly unknown. More than 5 million people are suffering from IPF and no definite treatment had been suggested; generally, it leads to death within 3 years of diagnosis (Antoniou et al., 2007 ▶). Pulmonary fibrosis is one of the disabling diseases which are not easily curable with common therapeutic methods, although available approaches may slow disease progression. Thus, finding new treatments is crucial for management of pulmonary fibrosis. Pulmonary fibrosis is characterized by extracellular matrix deposition in terminal airspacedue to severe or chronic pulmonary injury (Crouch, 1990 ▶; Garantziotis et al., 2004 ▶; Thannickal et al., 2004 ▶). Researches have shown that BLM induces pulmonary fibrosis in animals (Tzurel et al., 2002 ▶; Wang et al., 1991 ▶). Histopathological changes reveal that BLM-induced pulmonary injury includes two phases: 1) Premature inflammation phase characterized by leukocyte infiltration; and 2) Fibrotic phase characterized by collagen deposition and extracellular matrix deformation (Gao et al., 2011 ▶; Liang et al., 2011 ▶).

 As stated earlier, oxidative stress is among the main mechanisms involved in the pathogenesis of pulmonary fibrosis (Kinnula et al., 2005 ▶). Oxidative stress is defined as an imbalance between the production of reactive oxygen species (ROS) or free radicals and the antioxidant defense (Ceretta et al., 2012 ▶). Evidence shows that both inflammation (Bringardner et al., 2008 ▶; Homer et al., 2011 ▶) and oxidative stress (Kinnula et al., 2005 ▶; Walters et al., 2008 ▶) play important roles in the pathogenesis of pulmonary fibrosis. Of course, inflammation increases oxidative stress because of the accumulation of inflammatory cells such as macrophages and neutrophils in the lower respiratory tract that leads to increased oxidative stress (Crystal et al., 1984 ▶). For example, pulmonary inﬂammatory cells of IPF patients generate higher levels of oxidants than those of the control group (Cantin et al., 1987 ▶). On the other hand, pulmonary antioxidants-like glutathione are decreased in these patients (Cantin et al., 1989 ▶).

 Therefore, herbal medicines having anti-inflammatory and antioxidant properties may help to reduce oxidative stress and cause beneficial effect against pulmonary fibrosis (Samareh Fekri et al., 20015 ▶) . In this regard, *N. sativa *might be an effective treatment for pulmonary fibrosis and inflammation due to its above-mentioned properties (Ashraf et al., 2011 ▶; Ghannadi et al., 2005 ▶).

*N. sativa *is rich in phenolic compounds with antioxidants properties (Meziti et al., 2012 ▶). It also includes pharmacologically active compound, thymoquinone (Ghosheh et al., 1999 ▶) which is known to have antioxidant effects (Badary et al., 2003 ▶). Antioxidant effects of whatdepend on ability to trap and eliminate free radicals with production stable phenoxyl compounds (Lam et al., 2007 ▶). *N. sativa*has a protective role against oxidative stress and possesses scavenging activity for elimination of free radicals* (*Leong et al., 2013 ▶). Experimental studies in animal models indicated that BLM induces oxidative stress through generation of free radicals leading to fibrotic changes in the pulmonary parenchyma similar to that seen in IPF patients (Moeller et al., 2006 ▶). In some studies, it was demonstrated that feining and curcumin reduce BLM-induced inflammatory and oxidant activity by their polyphenolic moieties using their antioxidant effects (Liang et al., 2011 ▶; Venkatesan et al., 1997 ▶).

Other studies also indicated similar results which confirm the findings of our study. In a study, preventive effect of *N. sativa* extract on lung inflammation was investigated in sensitized guinea pigs. The results indicated a decrease in pathological changes of the lung and levels of inflammatory mediators such as IL-4 in bronchoalveolar lavage fluid (Boskabady et al., 2011a ▶).

 In another study, *N. sativa *showed a relaxant effect on guinea pig tracheal chains and this effect was significantly higher than that of theophylline. In addition, pretreatment of animals with *N. sativa*reduced tracheal responsiveness to cigarette smoke (Keyhanmanesh et al., 2013 ▶; Keyhanmanesh et al., 2014b ▶).

 The use of *N. sativa* extract at different concentrations improved the spirometry parameters in asthmatic patients similar to theophylline. Moreover, clinical symptoms namely, wheezing and recurrent attacks of asthma were reduced in patients treated with *N. sativa* compared to control group (Boskabady et al., 2007 ▶; Boskabady et al., 2010 ▶).

 In the present study, effect of methanolic extract of *N. sativa* on early and late prevention of BLM-induced pulmonary fibrosis was examined in rats. The results of this study showed that *N. sativa *extract significantly reduced pulmonary fibrosis and inflammation and this effect was more marked than that of M-pred (a known anti-inflammatory drug). Moreover, this plant indicated a significant reduction in hydroxyproline levels, an index of collagen deposition and pulmonary fibrosis, similar to M-pred. The effect of *N. sativa* on oxidative stress through decrease production peroxidation and increasing catalase activity may also support its effect in treatment of diseases such as pulmonary fibrosis in which free radicals play an important role. 

The present study suggest that *N. sativa *is effective in early and late prevention of pulmonary inflammation and fibrosis. However, more studies are needed for identification of its anti-inflammatory and anti-fibrotic mechanisms in the respiratory system. Therefore, it can be concluded that herbal medicines such as *N. sativa *that contain phenolic compounds, may possess therapeutic potentials in treatment of pulmonary fibrosis and inflammation. 
